# LSK Derived LSK^–^ Cells Have a High Apoptotic Rate Related to Survival Regulation of Hematopoietic and Leukemic Stem Cells

**DOI:** 10.1371/journal.pone.0038614

**Published:** 2012-06-04

**Authors:** Cong Peng, Yaoyu Chen, Yi Shan, Haojian Zhang, Zhiru Guo, Dongguang Li, Shaoguang Li

**Affiliations:** 1 Division of Hematology/Oncology, Department of Medicine, University of Massachusetts Medical School, Worcester, Massachusetts, United States of America; 2 School of Computer and Security Science, Edith Cowan University, Mount Lawley, Western Australia, Australia; Chang Gung University, Taiwan

## Abstract

A balanced pool of hematopoietic stem cells (HSCs) in bone marrow is tightly regulated, and this regulation is disturbed in hematopoietic malignancies such as chronic myeloid leukemia (CML). The underlying mechanisms are largely unknown. Here we show that the Lin^−^Sca-1^+^c-Kit^-^ (LSK^−^) cell population derived from HSC-containing Lin^−^Sca-1^+^c-Kit^+^ (LSK) cells has significantly higher numbers of apoptotic cells. Depletion of LSK cells by radiation or the cytotoxic chemical 5-fluorouracil results in an expansion of the LSK^−^ population. In contrast, the LSK^−^ population is reduced in CML mice, and depletion of leukemia stem cells (LSCs; BCR-ABL-expressing HSCs) by deleting *Alox5* or by inhibiting heat shock protein 90 causes an increase in this LSK^−^ population. The transition of LSK to LSK^−^ cells is controlled by the *Icsbp* gene and its downstream gene *Lyn*, and regulation of this cellular transition is critical for the survival of normal LSK cells and LSCs. These results indicate a potential function of the LSK^−^ cells in the regulation of LSK cells and LSCs.

## Introduction

Hematopoietic stem cells (HSCs) are contained in the LSK cell population are responsible for producing variable types of mature blood cell lineages. However, little is known about whether and how LSK cells are functionally regulated by other cell lineages that are derived from LSK cells and express similar cell surface markers. Phenotypically, LSK cells express Sca-1 and c-Kit, but lack the lineage (Lin) markers expressed on mature myeloid and lymphoid cells [1], [2], [3], [4]. The HSC-containing Lin^−^Sca-1^+^c-Kit^+^ (LSK) cell population is capable of self-renewing and differentiating into a c-Kit-negative population (Lin^−^Sca-1^+^c-Kit^−^, LSK^−^) that lacks the ability to reconstitute lethally irradiated mice [5], indicating that LSK^−^ cells do not have long-term stem cell reconstitution capability. Paradoxically, this cell population is shown to contain distinctive lymphoid progenitor cells [6], suggesting that LSK^−^ cells have some stem cell-like functions. This cell population also appears to be related to the development of a hematologic malignancy derived from leukemia stem cells (LSCs). For example, in mice with BCR-ABL induced chronic myeloid leukemia (CML), BCR-ABL-expressing LSK cells function as LSCs [7], and besides BCR-ABL-expressing LSK cells, the bone marrow of CML mice also contains BCR-ABL-expressing LSK^−^ cells [8], [9], although transplantation of cells containing the LSK^−^ cell population does not induce CML in secondary recipient [7]. Together, these results prompted us to hypothesize that although LSK^−^ cells do not function as HSC-containing LSK cells or LSCs, they play a critical role in regulating the survival of these stem cells in both normal and malignant situations through unknown mechanisms. Proper regulation of the LSK population is crucial for normal blood development [10], [11], [12]. In CML, improper regulation of LSCs occurs, resulting in an increase in the LSC population [8], [13]. It will be important to study the regulatory mechanisms responsible for survival and maintenance of normal LSK cells and LSCs. In this study, we demonstrate that LSK cells and LSCs in CML regulate their survival through becoming a more apoptotic cell population and that this cellular transition is controlled by the *Icsbp* gene and its downstream gene *Lyn*.

## Results

### LSK derived LSK^−^ cell population is related to survival regulation of LSK cells

**Figure 1:**
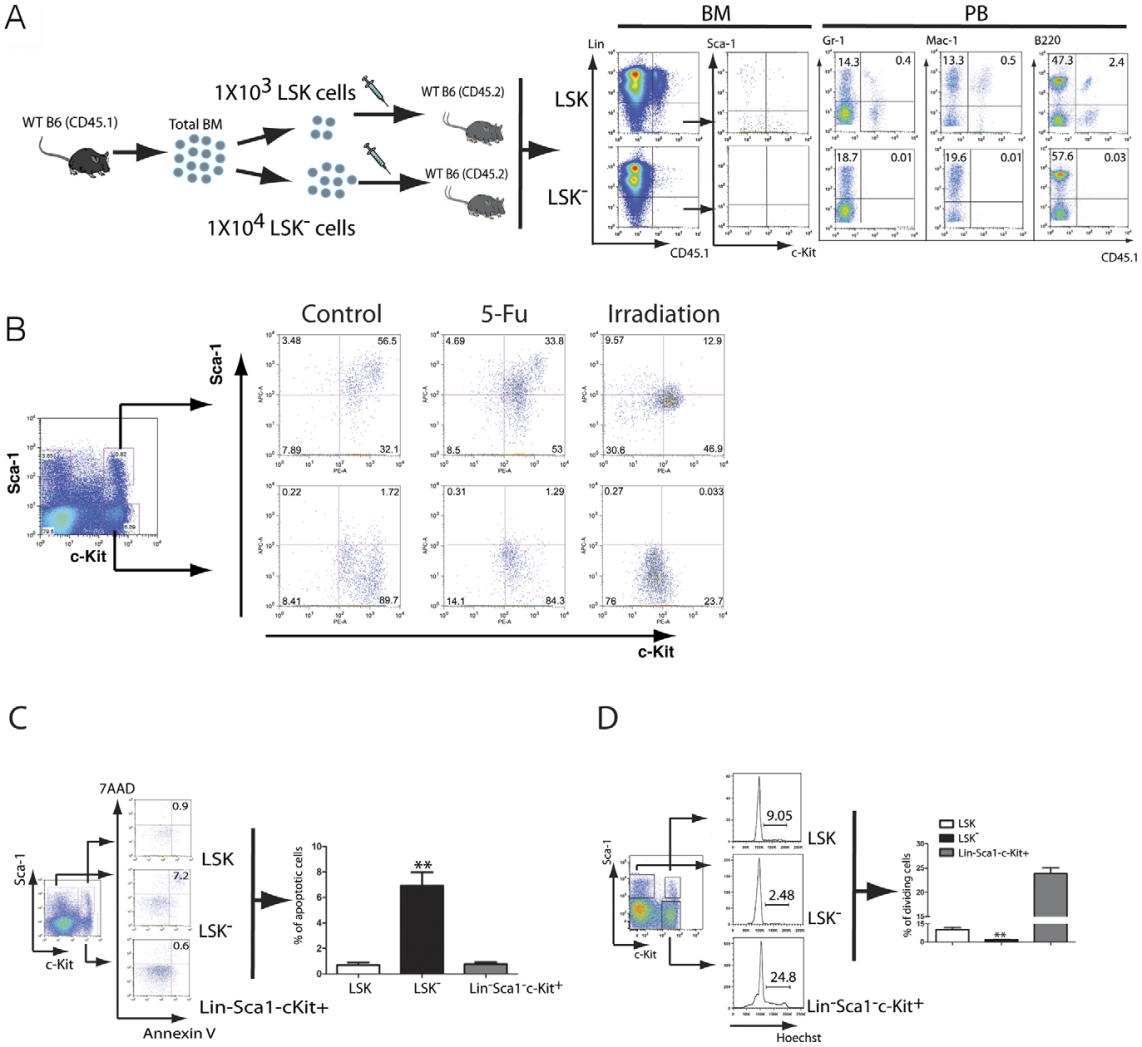
The LSK^−^ cell population is derived from LSK cells and provides an apoptotic cellular pathway for LSK cells. (**A**) LSK (Lin^−^Sca1^+^c-Kit^+^) cells (1×10^3^) and LSK^−^ (Lin^−^Sca1^+^c-Kit^−^) cells (1×10^4^) were sorted from bone marrow cells of CD45.1^+^ wild type (WT) mice by FACS, and transferred into lethal irradiated CD45.2^+^ WT recipient mice. 12 weeks later, bone marrow cells were collected and stained with antibodies for CD45.1, lineage markers, Sca−1 and c-Kit. Peripheral blood cells were also collected and stained with antibodies for CD45.1, Gr-1, Mac−1 and B220. (**B**) LSK cells were sorted from bone marrow of C56BL/6 (B6) mice by FACS and were irradiated (2000 cGy, once) or treated with 5-FU *in vitro*. The cells were cultured for 24 hours and then analyzed by FACS. (**C**) Apoptotic rates of progenitor and stem cells in vivo. Progenitor and stem cells were labeled with 7AAD and Annexin V, and analyzed by FACS (n = 5). **: *p*<0.01. (**D**) Cell cycle analysis of progenitor and stem cells in vivo. The cells were stained with Hoechst Blue, and the cells in the S+G2M phase were analyzed by FACS (n = 5). **: *p*<0.01.

Because LSK^−^ cells are derived from LSK cells and lack the ability to reconstitute lethally irradiated mice [5], we reasoned that this cell population is incapable of giving rise to LSK cells, progenitor cells and more mature cell lineages. To provide more supporting evidence, we transferred 1×10^4^ LSK^−^ cells or 1×10^3^ LSK cells (as a positive control) from CD45.1 C57BL/6J (B6) mice into lethally irradiated CD45.2 B6 recipients. Three months after the bone marrow transplantation, FACS analysis showed that donor-derived CD45.1 LSK and LSK^−^ cells were detected in bone marrow of recipients of CD45.1 LSK cells as expected, but neither cell population was detected in bone marrow of recipients of CD45.1 LSK^−^ cells (Fig. 1A). In addition, no donor-derived mature myeloid (Gr1^+^CD45.1^+^ and Mac1^+^CD45.1^+^) and B-lymphoid cells (B220^+^CD45.1^+^) were detected in peripheral blood of recipients of CD45.1 LSK^−^ cells (Fig. 1A). These results indicate that the LSK^−^ cell population lacks long-term stem cell reconstitution function or an ability to give rise to LSK cells, consistent with the failure of LSK^−^ cells to reconstitute lethally irradiated mice [5]. Although LSK cells give rise to LSK^−^ cells, we wanted to know whether the LSK^−^ cells could be directly differentiated from LSK or from other lineage negative populations. First, we sorted LSK cells from bone marrow of B6 mice, and treated the cells with the cytotoxic agent 5-FU or irradiation *in vitro*. The cells were then cultured for examining whether LSK^−^ population could be produced directly from LSK cells. After the treatment with irradiation or 5-FU, we detected higher percentages of LSK^−^ cells compared to the untreated control (Figure 1B). Second, we similarly treated Lin^−^Sca-1^-^c-Kit^+^ (LS^−^K; representing progenitor cells) with 5-FU or irradiation, and barely detected any LSK^−^ cells (Fig. 1B), indicating that LS^−^K cells do not give rise to LSK^−^ cells. Finally, we tested whether LSK^−^ cell could arise from Lin^−^Sca-1^-^c-Kit^−^ (LS^−^K^−^) cells by carrying out an *in vivo* reconstitution experiment. We transplanted 1×10^6^ CD45.1 LS^−^K^−^ cells into each CD45.2 recipient mouse, and continuously monitored CD45.1 donor cells in the recipient mice at 1, 2, 4, 8 and 16 weeks post bone marrow transplantation (BMT). At the first week, small percentages of CD45.1 cells were detected (Fig. S1A), but with time, CD45.1 cells disappeared in CD45.2 recipient mice (Fig. S1A, S1B), indicating that LS^−^K^−^ cells do not give rise to any other populations including LSK^−^ cells.

It is possible that the transition of LSK cells to LSK^−^ cells provides a cellular mechanism for regulating the LSK population. To test this idea, we compared apoptotic rates of three LSK derived cell populations in bone marrow: LSK, LSK^−^ and LS^−^K. By FACS analysis of Annexin V^+^/7-AAD^+^ cells, we observed that apoptotic rates of LSK and LS^−^K cells were low (0.9% and 0.6% in average, respectively), but apoptotic rate of LSK^−^ cells was much higher (7.2% in average) (Fig. 1C). We also compared the percentages of dividing cells in these three LSK derived cell populations in bone marrow, and found that the percentage of LSK^−^ cells in the S+G_2_M phase was much lower than those in the other two populations (Fig. 1D). These results indicate that the LSK^−^ population represents a pool of resting and apoptotic cells.

**Figure 2:**
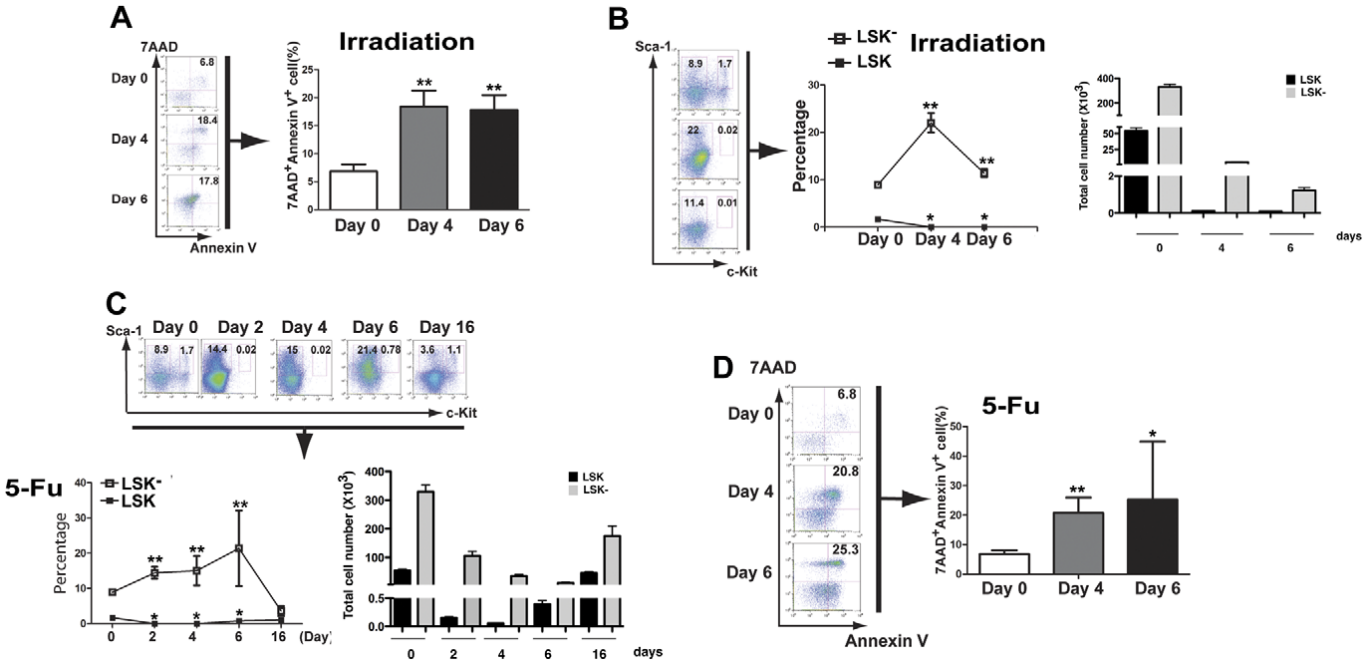
Irradiation and 5-FU treatment promote cellular transition of LSK to LSK^−^ cells. (**A**) Lethal irradiation causes an increase in the percentage of apoptotic LSK^−^ cells. Mice were treated by two split doses of 550-cGy gamma irradiation (separated by 3 hours), and the apoptotic cells (7AAD^+^Annexin V^+^) in the LSK^−^ population were analyzed by FACS at different time points (n = 4). **: *p*<0.01. (**B**) Total number and percentages of LSK and LSK^−^ cells in bone marrow of lethally irradiated WT mice were determined at different time points (n = 4). *: *p*<0.05; **: *p*<0.01. (**C**) Total number and percentages of LSK and LSK^−^ in bone marrow of 5-FU treated WT mice at different time points. The mice were treated with 5-FU (200 mg/kg) by intravenous injection, and the percentages of LSK and LSK^−^ cells were monitored at different time points (n = 4). *: *p*<0.05; **: *p*<0.01. (**D**) The apoptotic rate of LSK^−^ cells in 5-FU treated mice. WT mice were treated with 5-FU, and apoptotic rate of LSK^−^ cells were monitored at different time points (n = 4). *: *p*<0.05; **: *p*<0.01.

Because LSK^−^ cells are derived from LSK cells [5] and incapable of giving rise to LSK cells (Fig. 1A), it is possible that LSK^−^ cells regulates LSK cells through providing an apoptotic cellular pathway to regulate the pool size of the LSK population through controlling the degree of the transition of LSK cells to more apoptotic LSK^−^ cells. To test this hypothesis, we examined whether induction of apoptosis of LSK cells is associated with an increase in the LSK^−^ population *in vivo*. First, we lethally irradiated B6 mice, and found that the irradiation induced apoptosis of LSK cells in bone marrow (Fig. 2A), which accompanied with a decrease of LSK cells and an increase of LSK^−^ cells (Fig. 2B). Second, we treated B6 mice with 5-FU, which has been shown to reduce LSK cells within initial two days of the treatment [14], and found that apoptosis of LSK cells also accompanied with a decrease of LSK cells and an increase of LSK^−^ cells (Fig. 2C, D).

### An increase in LSK^−^CD150^-^ cells reflects apoptosis of LSK cells

**Figure 3:**
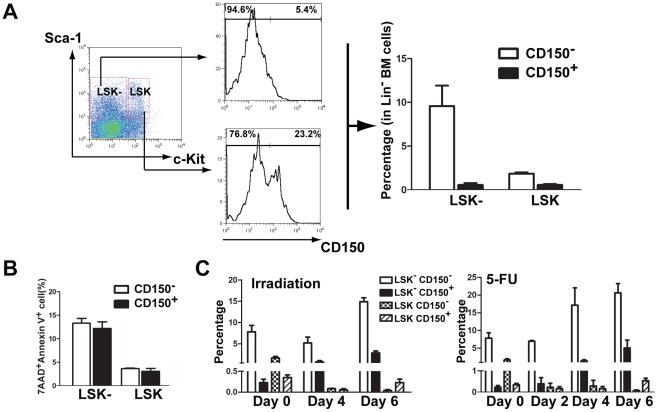
An increase in LSK ^−^ CD150^−^ cells reflects apoptosis of HSCs. (**A**) The LSK^−^ cell population is basically CD150-negative. LSK^+^ (Lin^−^Sca1^+^c-Kit^+^) cells and LSK^−^ (Lin^−^Sca1^+^c-Kit^−^) cells from bone marrow of B6 mice were stained with CD150, and analyzed by FACS. (**B**) High apoptotic rates in bone marrow LSK^−^CD150^−^ cells. LSK^+^ and LSK^−^ cells from bone marrow of B6 mice were stained with CD150, further labeled with 7AAD and Annexin V, and analyzed by FACS. Apoptotic rate for each cell population was indicated. (**C**) An increase in LSK^−^CD150^−^ cells reflects apoptosis of HSCs. B6 mice were treated with irradiation or 5-FU for different days as indicated. LSK^+^ and LSK^−^ cells from bone marrow of B6 mice were stained with CD150, were further labeled with 7AAD and Annexin V, and analyzed by FACS.

CD150 is a major SLAM family receptor expressed on HSCs and has been shown to be a useful cell surface marker for classifying LSK cells into a highly enriched stem cell population [15]. To more clearly show the relationship between the apoptotic LSK^−^ cell population and HSCs, we further narrowed down the LSK and LSK^−^ populations by adding CD150 analysis, and re-validated our 5-FU and irradiation experiments shown in Fig. 2. We found that in normal B6 mice, about 1/4 (23.2%) of bone marrow LSK cells in the lineage-negative (Lin^−^) population were CD150 positive and 3/4 (76.8%) of these cells were CD150 negative (Fig. 3A); both cell populations had similar apoptotic rates (3.4% and 3.5%; Fig. 3B). In contrast, greater than 90% the LSK^−^ cells were CD150^−^, with only about 5.4% of CD150^+^ cells; both populations also had similar apoptotic rates (13% and 14%; Fig. 3B), which were much higher than those for the LSK cells. Thus, the addition of CD150 staining to LSK^+^ and LSK^−^ cells gave the same result as the one obtained without using the CD150 marker (Fig. 2A, D). In addition, because almost all cells in the LSK^−^ population are CD150^−^ (Fig. 3A), the addition of CD150 staining would not change the result for the apoptotic rate of this cell population. To further emphasize this point, we repeated our 5-FU and irradiation experiments (Fig. 2) by adding the CD150 staining to LSK^+^ and LSK^−^ cells. Comparing to untreated mice (Day 0) in which LSK^−^ cells were basically negative for CD150 (about 95% of LSK^−^ cells were CD150^−^), both treatments caused a remarkable increase in the LSK^−^CD150^−^ population as expected and also surprisingly in the LSK^−^CD150^+^ population, suggesting that a significant number of HSCs (LSK^+^CD150^+^) made a quick transition after irradiation or 5-FU treatment to become apoptotic LSK^−^ cells, presumably without having time to turn down CD150 expression. In contrast, in a normal situation there are few LSK^−^CD150^+^ cells (Fig. 3A). The increase of the LSK^−^CD150^−^ population after irradiation or 5-FU treatment accompanied with the decrease of the LSK^+^CD150^+^ population. This result, in addition to the fact that LSK^−^ cells are basically negative for CD150, demonstrates that an increase in the LSK^−^ population detected without using the CD150 marker should accurately reflect cellular transition of HSCs and LSCs to the LSK^−^ population and apoptosis of these stem cells. Therefore, we excluded the use of the CD150 marker in the rest of our experiments in this study.

### The LSK^−^ cell population is associated with apoptosis of LSCs

**Figure 4:**
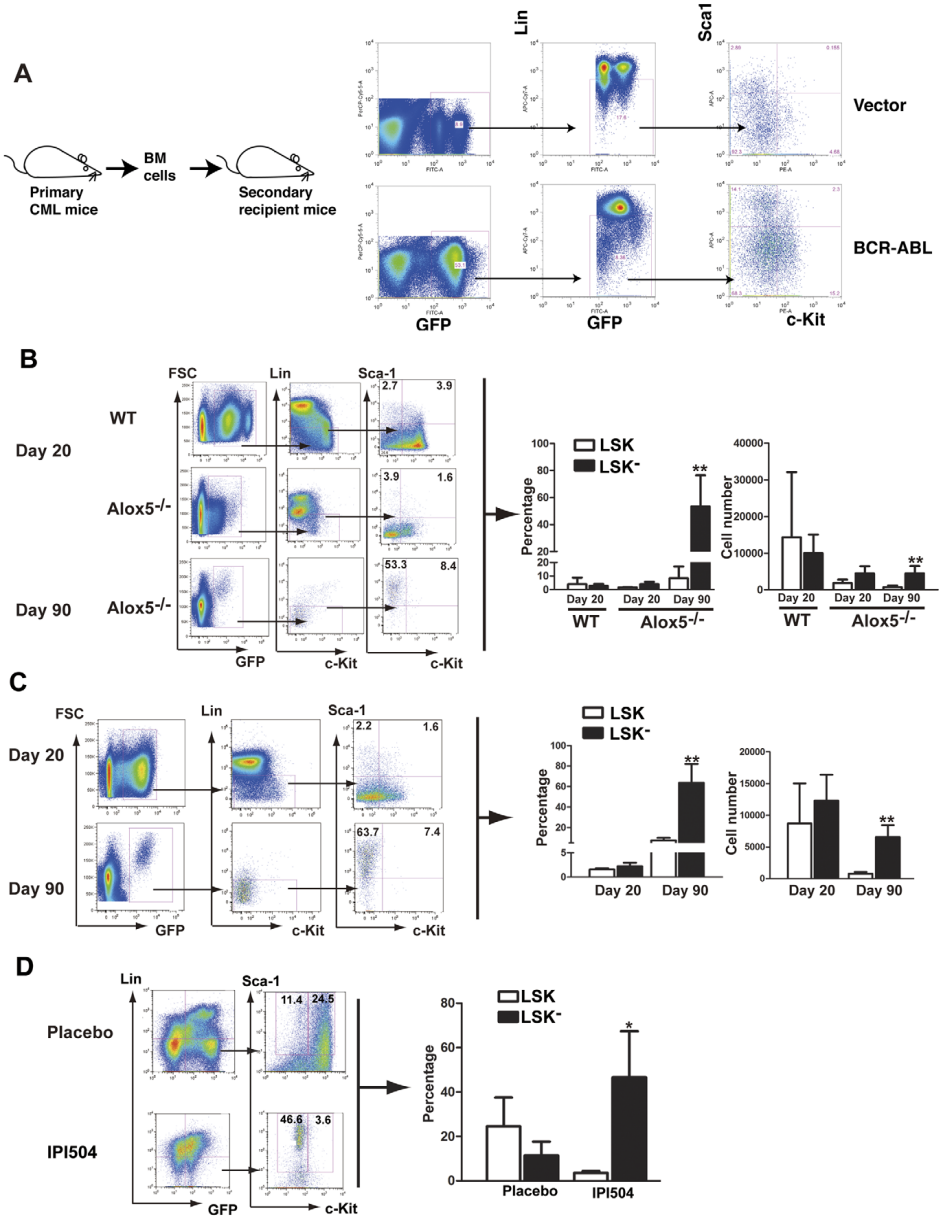
Suppression of LSCs through disturbing the *Alox5* or *Hsp90* pathways is associated with enhanced transition of LSCs to BCR-ABL-expressing LSK^−^ cells. (**A**) BCR-ABL^+^LSK cells give rise to LSK^−^ cells in CML mice. 1×10^6^ bone marrow cells (containing BCR-ABL^+^LSK cells) from primary CML mice were transplanted into each secondary recipient mouse. 15 days later, bone marrow cells were analyzed by FACS for the presence of BCR-ABL^+^LSK and LSK^−^ cells. (**B**) *Alox5* deletion enhanced the transition of LSCs (GFP^+^LSK) to GFP^+^ LSK^−^ cells. At day 20 after induction of CML, the percentage of LSCs and GFP^+^ LSK^−^ cells in bone marrow of recipients of BCR-ABL transduced WT donor marrow cells were determined (top panel), and the percentage of GFP^+^LSK^−^ cells was lower than that of LSCs. In recipients of BCR-ABL transduced *Alox5^−/−^* donor marrow cells, the percentage of GFP^+^ LSK^−^ cells became higher than that of LSCs (middle panel). At day 90, the percentage of GFP^+^LSK^−^ cells reached as high as 50% (bottom panel) (n = 4 for each group). **: *p*<0.01. (**C**) Inhibition of *Alox5* function by Zileuton enhanced the transition of LSCs to BCR-ABL expressing Lin^−^Sca1^+^c-Kit^−^ cells. CML mice were treated with Zileuton (300 mg/kg body weight, twice a day) for 90 days and the percentages of LSCs and GFP^+^LSK^−^ were monitored by FACS (n = 4 for each group). **: *p*<0.01. (**D**) The percentages of LSCs and BCR-ABL-T315I expressing Lin^−^Sca1^+^c-Kit^−^ cells were measured by FACS after IPI-504 treatment. Bone marrow cells from CML mice were cultured under a stem cell culture condition in the presence of IPI-504 (100 nM) for 6 days, and the percentages of LSCs and GFP^+^LSK^−^ were determined by FACS. *: *p*<0.05.

If LSK^−^ cell population represents an apoptotic regulatory pathway for LSK cells, in a hematologic malignancy the transition of malignant LSK cells to apoptotic LSK^−^ cells could be blocked. To test this possibility, we first intended to show that in CML mice, leukemia stem cells (LSC; BCR-ABL^+^ LSK cells) also undergo transition to BCR-ABL^+^LSK^−^ cells. Because only BCR-ABL^+^LSK cells are capable of self-renewing and initiating leukemia in secondary recipient mice, transferring LSC-containing bone marrow cells in CML mice to secondary recipient mice allows examination of the transition of BCR-ABL^+^LSK to BCR-ABL^+^LSK^−^ cells. To induce primary CML, we transduced bone marrow cells from wild type (WT) B6 mice with BCR-ABL-GFP retrovirus (GFP serves as an indicator for BCR-ABL expression), followed by transplantation of the transduced cells into lethally irradiated B6 recipient mice [9], [16]. Next, we transplanted 1×10^6^ total bone marrow cells from primary CML mice to each secondary recipient mouse, and the mice developed CML and died of CML within 4 weeks (Fig. S2A, S2B). After 15 days of secondary transplantation, we analyzed the GFP^+^LSK and LSK^−^ populations in bone marrow of the secondary CML mice, and detected by FACS both BCR-ABL^+^LSK and LSK^−^ cells, demonstrating that the LSK to LSK^−^ transition also occurs for LSCs (Fig. 4A). This finding allowed us to further study the role of LSK^−^ cells in regulation of LSCs. We first examined whether the percentage of bone marrow LSK^−^ cells is decreased in mice with BCR-ABL induced CML. We found that the average percentage of GFP^+^LSK^−^ cells in CML mice at 20 days after the disease induction was significantly lower (2.7%; Fig. 4B) than that of GFP^-^LSK^−^ cells (7.4% in average; data not shown) and that of LSK^−^ cells in normal B6 mice (8.9%; Fig. 2B). To provide more evidence supporting the role of GFP^+^LSK^−^ cells in regulating apoptosis of LSCs in CML, we induced CML using the BCR-ABL transduced bone marrow cells from mice lacking the *Alox5* gene (*Alox5* ^−*/−*^), as in the absence of *Alox5*, BCR-ABL fails to induce CML due to a functional defect in *Alox5^−/−^* LSCs [17]. We compared the number of GFP^+^LSK^−^ cells in bone marrow of mice receiving BCR-ABL transduced *Alox5^−/−^* donor bone marrow cells with that in mice receiving BCR-ABL transduced WT donor cells. We found that the *Alox5* deficiency caused a significant increase in the percentage of bone marrow GFP+LSK- cells (Fig. 4B). Consistent with this finding, CML mice treated with Zileuton, which reduces survival of CML LSCs by inhibiting the function of 5-lipoxygenase (the *Alox5* gene product) [17], had a marked increase in bone marrow GFP^+^LSK^−^ cells (Fig. 4C). The increase in LSK^−^ cells explains the depletion of LSCs in CML mice treated with Zileuton [17], which could be a general mechanism utilized by chemical compounds that suppress LSCs. To strengthen this idea, we examined whether LSK^−^ cells are also increased in CML mice treated with a HSP90 inhibitor, IPI-504, which has been shown to inhibit LSCs in CML mice [18]. In this experiment, we used BCR-ABL-T315I to induce CML in mice, because we previously showed that compared to wild type BCR-ABL, CML cells harboring BCR-ABL-T315I in mice were more dependant on HSP90 for stability and therefore BCR-ABL-T315I protein was more sensitive to HSP90 inhibition for degradation [9], providing a more sensitive assay for testing the transition of LSCs to LSK- cells. We found that IPI-504 treatment caused a marked increase in LSK^−^ cells compared with the CML mice treated with a placebo (Fig. 4D). These results demonstrate that the transition from LSK cells or LSCs to apoptotic LSK^−^ cells provides a cellular pathway for regulating these two cell populations.

### Icsbp and Lyn regulate the transition of LSK to LSK^−^ cells

**Figure 5:**
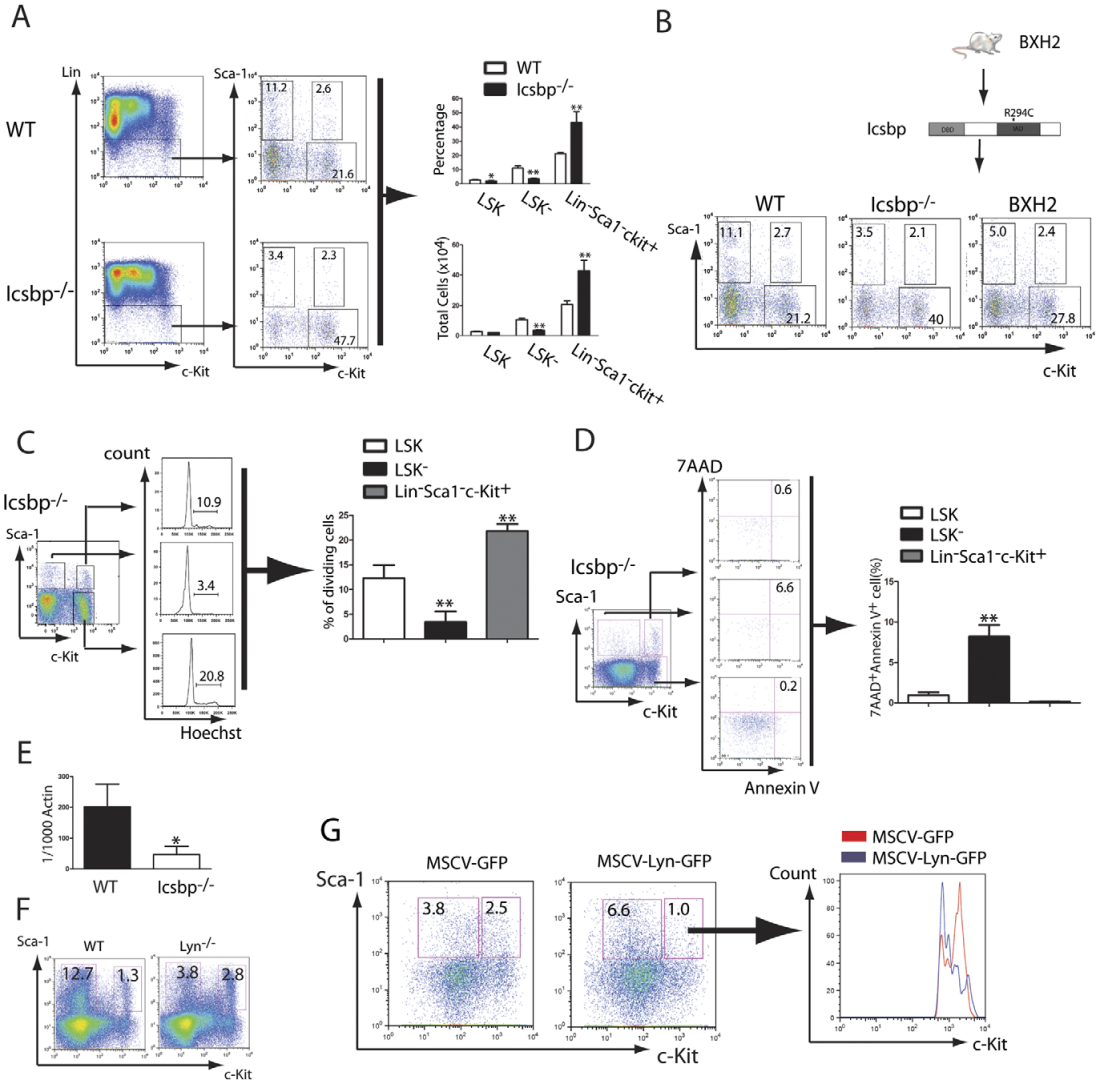
The *Icsbp*-*Lyn* pathway controls the transition of LSK to LSK^−^ cells. (**A**) The percentages and numbers of LSK, LSK^−^ and Lin^−^Sca1^−^c-Kit^+^ were compared between WT and *Icsbp^−/−^* mice (n = 4 for each group). *: *p*<0.05; **: *p*<0.01. (**B**) The percentages of LSK, LSK^−^ and Lin^−^Sca1^−^c-Kit^+^ were compared between WT and BXH2 mice. (**C**) Cell cycle analysis of progenitor and stem cells in *Icsbp^−/−^* mice. Bone marrow cells were stained with Hoechst blue, and DNA contents, represented by the percentages of progenitor and stem cells in the S+G2M phase of the cell cycle, were examined by FACS (n = 4). **: *p*<0.01. (**D**) Apoptotic rates of progenitor and stem cells in *Icsbp^−/−^* mice. The cells were labeled with 7AAD and Annexin V, and the percentages of progenitor and stem cells were determined by FACS (n = 4). **: *p*<0.01. (**E**) *Lyn* expression in bone marrow cells was compared between WT and *Icsbp^−/−^* mice. Bone marrow cells were collected from WT and *Icsbp* ^−/−^ mice, respectively, and *Lyn* expression was detected by real-time PCR (n = 3). *: *p*<0.05. (**F**) The percentages of LSK and LSK^−^ were compared between WT and *Lyn^−/−^* mice. *Lyn* deletion caused an increase in the percentage of LSK and decrease in the percentage of LSK^−^ cells. (**G**) Overexpression of *Lyn* caused an increase in the LSK^−^ population. *Lyn* ^−/−^ bone marrow cells were transduced with *MSCV-GFP* or *MSCV-Lyn-GFP* retrovirus, followed by transplantation of the transduced cells into lethally irradiated WT recipient mice. At day 60 after the transplantation, the percentages of LSK and LSK^−^ cells were determined by FACS.

To study the underlying mechanisms for the transition between LSK and LSK^−^ cells, we decided to focus on the interferon consensus sequence binding protein (*Icsbp*) gene, because mice lacking *Icsbp* (*Icsbp^−/−^*) develops CML-like disease [19]. Our DNA microarray study showed that *Icsbp* was downregulated by BCR-ABL in LSCs and this downregulation was partially restored in *Alox5^−/−^* LSCs (Fig. S3). This result was confirmed by RT-PCR (Fig. S4). Expression of *Icsbp* is also down-regulated in CML patients [20]. Furthermore, we found that the percentage of LSK^−^ cells in bone marrow of *Icsbp^−/−^* mice (8 weeks old) was dramatically lower than that in WT mice (Fig. 5A), suggesting that the normal function of *Icsbp* is to promote the transition of LSK cells to LSK^−^ cells. A plausible explanation for the development of CML-like disease in *Icsbp^−/−^* mice is that when *Icsbp* is removed, LSK cells could not undergo the transition to apoptotic LSK^−^ cells, and the LSK population could only be regulated through differentiating to become more mature myeloid cells, eventually leading to the development of CML-like disease, as observed in *Icsbp^−/−^* mice [19]. Consistent with this idea, the percentage and total number of LSK^−^ cells in bone marrow of old *Icsbp^−/−^* mice (24 weeks old) was much lower than that in old WT mice (Fig. S5). The function of *Icsbp* in controlling the transition of LSK to LSK^−^ cells was also demonstrated in BXH2 mice, in which the function of *Icsbp* is impaired by a R294C mutation, leading to an eventual development of CML-like disease [21]. We observed that similar to *Icsbp^−/−^* mice, BXH2 mice had a lower percentage of LSK^−^ cells than did WT mice (Fig. 5B). We then examined the percentages of dividing and apoptotic cells in *Icsbp^-/-^* mice (Fig. 5C, D), and found that LSK^−^ cells in *Icsbp^−/−^* mice were relatively apoptotic and quiescent, similar to the LSK^−^ cells in WT mice (Fig. 2B, C). These results indicate that *Icsbp* does not regulate cell cycle and apoptosis of LSK^−^ cells but controls the transition of LSK to LSK^-^ cells.

Our finding of the role of *Icsbp* in the regulation of LSK transition prompted us to further investigate this *Icsbp* genetic pathway. Lyn tyrosine kinase plays an essential role in regulating hematopoiesis. *Lyn*-deficient mice display a collection of hematopoietic defects, including autoimmune disease as a result of autoantibody production, and perturbations in myelopoiesis that ultimately lead to splenomegaly and myeloid neoplasia, similar to CML-like disease observed in *Icsbp^−/−^* mice [22]. Induction of *Icsbp* expression in the *Icsbp^−/−^* myeloid progenitor cells leads to an upregulation of *Lyn* expression [23]. Therefore, we tested whether *Lyn* is involved in the regulation of the LSK population by *Icsbp* through controlling the transition of LSK to LSK^−^ cells. We first compared the mRNA levels of *Lyn* in bone marrow between WT and *Icsbp^−/−^* mice by RT-PCR, and found that *Lyn* was downregulated in *Icsbp^−/−^* bone marrow cells (Fig. 5E), suggesting that *Lyn* is downstream of *Icsbp*. We next determined whether the LSK^−^ cell population is reduced in *Lyn^−/−^* mice. Consistent with *Icsbp^−/−^* mice (Fig. 5A), the percentage of LSK^−^ cells in bone marrow of *Lyn^−/−^* mice was significantly reduced (Fig. 5F), demonstrating that *Lyn* is also involved in the transition of LSK to LSK^−^ cells. Furthermore, we tested whether expression of *Lyn* in bone marrow cells from *Lyn^−/−^* mice restores the cellular transition. We transduced *Lyn^−/−^* bone marrow cells with *MSCV-GFP* or *MSCV-Lyn-GFP* retrovirus, followed by transplantation of the transduced cells into recipient mice. The percentage of LSK^−^ cells in recipients of *MSCV-Lyn-GFP* transduced *Lyn^−/−^* bone marrow cells was about two-fold higher than that in recipients of *MSCV-GFP* transduced *Lyn^−/−^* bone marrow cells (6.59% vs. 3.79%) (Fig. 5G), supporting the role of *Lyn* as a downstream regulatory gene of *Icsbp* in maintaining the transition of LSK to LSK^−^ cells.

### The transition of LSCs to the LSK^−^ cell population is blocked in CML

**Figure 6:**
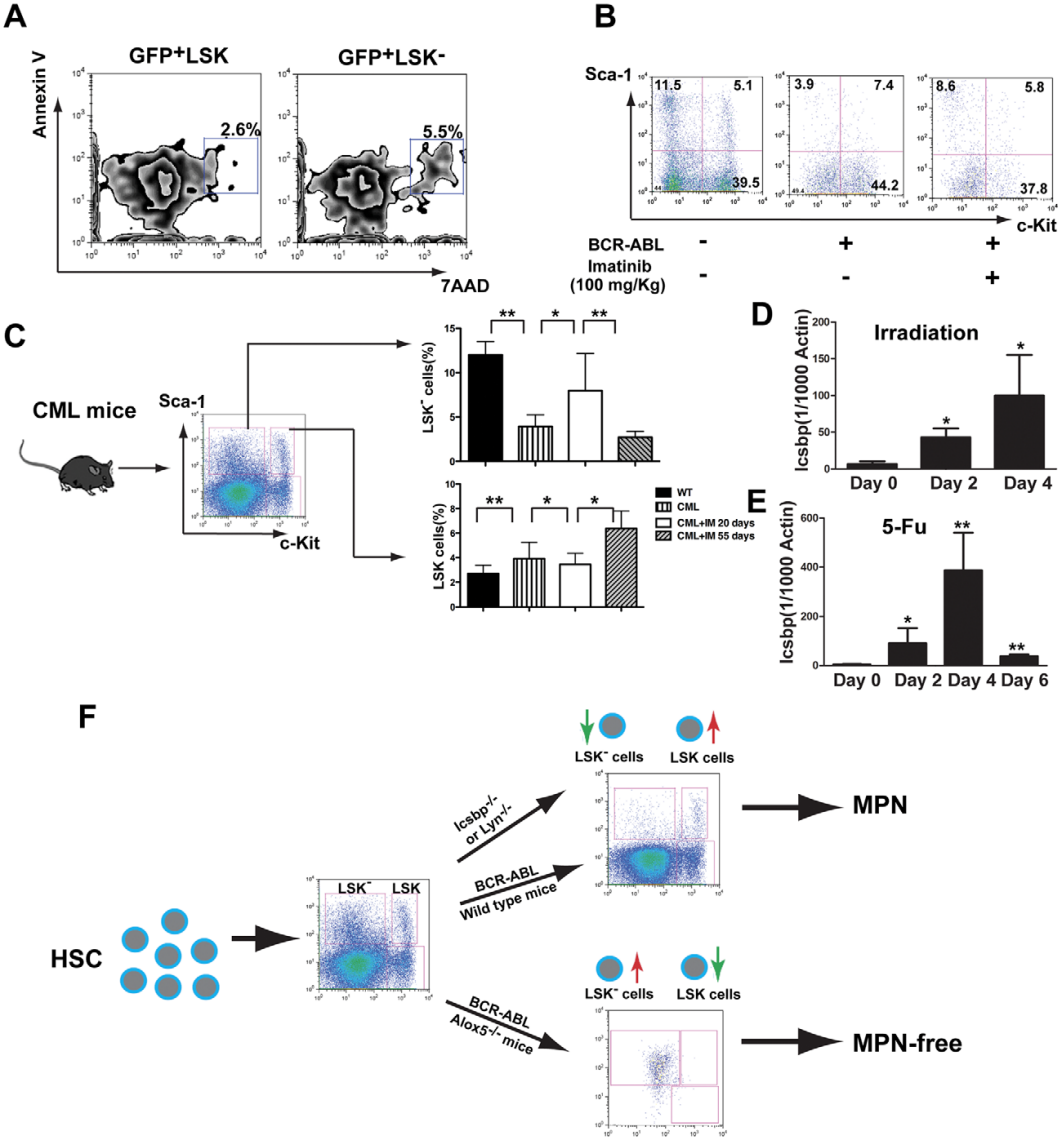
Long-term treatment of CML mice with imatinib fails to promote the transition of LSCs to BCR-ABL expressing LSK^−^ cells. (**A**) Apoptotic rates between LSCs and BCR-ABL expressing LSK^−^ cells in CML mice were compared. At day 14 after induction of CML, apoptotic rate in GFP^+^LSK^−^ was slightly higher than that of LSCs. (**B**) The percentage of GFP^+^LSK^−^ cells was increased after imatinib treatment for 12 days, beginning at day 8 after induction of CML (100 mg/kg). (**C**) The percentages of LSCs and GFP^+^LSK^−^ cells in bone marrow of CML mice were compared after imatinib treatment for 55 days. *: *p*<0.05; **: *p*<0.01. (**D**) *Icsbp* expression was increased in bone marrow cells of mice treated with lethal irradiation. Bone marrow cells were collected from lethally irradiated mice at day 0, 2 and 4 after irradiation. Total RNA was extracted and *Icsbp* expression was determined by real-time PCR. *: *p*<0.05. (**E**) *Icsbp* expression in bone marrow cells was determined in 5-FU treated mice. Bone marrow cells were collected from 5-FU treated mice at day 0, 2, 4 and 6 after the treatment, and total RNA was extracted and *Icsbp* expression was determined by real-time PCR. *: *p*<0.05; **: *p*<0.01. (**F**) Proposed molecular model for the control of the transition of LSK/LSCs and LSK^−^ cells. The balance between LSK and LSK^−^ cells is controlled by the *Icsbp*/*Lyn* pathway in normal hematopoiesis and by *Alox5* in BCR-ABL induced CML.

Because LSK^−^ cells in bone marrow of WT and *Icsbp^−/−^* mice are more apoptotic than LSK cells (Fig. 1C and Fig. 5D), we thought that this cellular pathway controlled by *Icsbp* might also play a role in regulating LSCs in CML mice. To test this idea, we first examined whether BCR-ABL-expressing LSK^−^ cells contain more apoptotic cells than LSCs in bone marrow of CML mice, and found that apoptotic cells in the GFP^+^LSK^−^ cell population were two-fold higher than those in the GFP^+^LSK cell population (Fig. 6A). We wondered whether the effect of imatinib treatment is also associated with the LSK^−^ cell population, and found that 20 days after CML induction, the percentage of LSK^−^ population was about 3.93% in placebo treated CML mice and 8.62% in imatinib treated CML mice (Fig. 6B), suggesting that the therapeutic effect of imatinib is associated with increased cellular transition of LSK cells. Because the BCR-ABL kinase inhibitor imatinib has an initial therapeutic effect but does not eliminate LSCs in CML mice [7], [17], we reasoned that the failure of imatinib to cure CML mice should be associated with an inability of imatinib to cause an increase in the transition of LSCs to LSK^−^ cells in CML mice treated for a long period of time. We observed that the percentage of LSK^−^ cells in bone marrow of imatinib-treated CML mice increased transiently, but eventually decreased to a level similar to that in untreated CML mice (Fig. 6C). This result suggests that LSCs somehow find a way to overcome imatinib-mediated inhibition of the transition of LSCs to apoptotic LSK^−^ cells.

The development of CML-like disease in *Icsbp^−/−^* mice prompted us to examine whether *Icsbp* regulates the transition of LSK cells to more apoptotic LSK^−^ cells during normal hematopoiesis. Because irradiation or 5-FU treatment causes a decrease in LSK cells and an increase in the apoptotic LSK^−^ cells (Fig. 2B, C), we detected *Icsbp* expression in bone marrow cells by real-time PCR under both conditions. We found that *Icsbp* expression levels were increased after irradiation (Fig. 6D) and 5-FU treatment (Fig. 6E), which were associated with the increase of LSK^−^ cells (Fig. 2B, C).

## Discussion

During normal hematopoiesis, HSCs progressively give rise to different progenitor cells, which in turn generate various mature cell lineages [24], [25], [26]. This process is precisely controlled to maintain each cell population. Homeostasis between the quiescent and activated states of stem cells is essential to balance stem cell maintenance with ongoing tissue regeneration [23]. In addition, HSCs and their lineage choices are tightly regulated by *Pten* to prevent AML initiation from HSCs [27], [28]. In CML development, normal control of hematopoiesis is largely disturbed at the level of HSCs [29], [30], [31], [32]. A complete study of the underlying mechanisms is critical for understanding how HSCs and LSCs survive and self-renew. In this study, we found that the LSK^−^ population derived from HSC-containing LSK cells does not have stem cell functions but is capable of regulating the survival of the LSK population by providing a cellular path for irreversible transition of LSK to highly apoptotic LSK^−^ cells. Activation of this apoptotic cellular pathway is normally maintained by expression of the *Icsbp*, *Lyn* and likely other related genes, and during myeloid transformation such as in CML, this pathway is shut down due to *Icsbp* downregulation. It is reasonable to speculate that when the number of LSK cells needs to be reduced to maintain the LSK population, the transition of LSK to apoptotic LSK^−^ cells would help to remove overly produced LSK cells. If this cellular pathway were blocked, LSK cells would be forced to differentiate, which would favor the development of myeloproliferative disease (Fig. 6F). This idea is supported by our observation that BCR-ABL causes an increase in the LSC population and that the defect in CML induction by BCR-ABL in the absence of *Alox5* is associated with increases in *Icsbp* expression and the percentage of BCR-ABL-expressing apoptotic LSK^−^ cells (Fig. 6F). In CML patients, *Icsbp* has been found to be downregulated [20]. Overexpression of *Icsbp* causes a delayed development of BCR-ABL induced CML in mice [33]. The maintenance of an active cellular pathway for apoptosis of LSCs by the *Icsbp* pathway provides an alternative explanation for the tumor suppressor role of *Icsbp* in CML development.

Comparing the LSK^−^ cells derived from normal HSCs and BCR-ABL-expressing LSK cells in CML, we did not observe any difference in the apoptotic rates. Thus, we believe that both normal HSCs and BCR-ABL^+^ LSK cells can similarly give rise to LSK^−^ cells. Because LSK^−^ cells can only arise from HSCs or BCR-ABL-expressing LSKs and have a much higher apoptotic rate, the transition to LSK^−^ cells would provide a regulatory mechanism for the survival of HSCs and BCR-ABL-expressing LSKs, which would ultimately affect normal hematopoiesis and leukemogenesis. On the other hand, because imatinib does not have a significant effect on CML stem cells, we would not expect that imatinib stimulates the transition of BCR-ABL-expressing LSK cells to LSK^−^ cells.

The molecular mechanism for maintaining and regulating the survival of the LSK population is poorly understood, and available evidence shows that it is a genetically-regulated process involving specific genes expressed in HSCs and bone marrow niche [10], [11], [12]. In this study, we provide evidence showing that the transition of HSC-containing LSK cells to apoptotic LSK^−^ cells is regulated by *Icsbp* and *Lyn*. It is reasonable to think that in myeloid proliferative diseases, expression of *Icsbp* and *Lyn* are inhibited, leading to a blockade of the transition of malignant LSK to LSK^−^ cells. This transition blockade would cause an increase in the malignant LSK. In support of this idea, we have shown that in CML, the BCR-ABL oncogene is expressed in LSK cells and causes an increase in this cell population [9], [13], and in this study we show that the transition blockade occurs in CML. It is likely that LSK cells communicate with LSK^−^ cells to mutually regulate, and *Icsbp* and *Lyn* are somehow involved. Although the underlying mechanisms are totally unknown, it is reasonable to think that cell-cell interaction and secreted cytokines could play roles in the regulation of this cellular transition. Further studies will help to answer these questions. Besides the *Icsbp*/*Lyn* pathway, it is highly possible that the transition of LSK to apoptotic LSK^−^ cells may also be regulated by other unknown mechanisms, and complete understanding of the molecular basis of these pathways will help to develop new strategies for targeting stem cells in the treatment of human diseases related to HSCs and LSCs.

## Methods

### Mice

C57BL/6J (B6) (CD45.2 positive), congenic B6 mice expressing CD45.1 (B6.SJL-*Ptprca Pepcb/BoyJ*), B6.129S2-*Alox5^tm1Fun^*/J (*Alox5^−/−^*), and B6.129S4-*Lyn^tm1Sor^*/J (*Lyn^−/−^*) mice were obtained from The Jackson Laboratory. *Icsbp^-/-^* mice were kindly provided by Dr. Herbert C. Morse (Laboratory of Immunopathology, NIAID, NIH). Mice were maintained in a temperature and humidity controlled environment and given unrestricted access to 6% chow diet and acidified water. All animal studies were approved by Institutional Animal Care and Use Committee.

### Bone marrow transduction/transplantation

The retroviral constructs *MSCV-GFP*, *MSCV-BCR-ABL-GFP* and *MSCV-Lyn-GFP* were used to make retroviral stocks by transient transfection of 293T cells using the kat system as previously described [34]. Six to ten week-old wild type and *Lyn^−/−^* mice were used for leukemogenesis and *Lyn* overexpression experiments. Induction of CML in mice was done as described previously^11^. Briefly, to induce CML, bone marrow cells from 5-FU (200 mg/kg) donor mice were transduced twice with BCR-ABL retrovirus by cosedimentation in the presence of IL-3, IL-6, and SCF. Wild type recipient mice were prepared by 1100 cGy gamma irradiation. A dose of 0.5×10^6^ (CML) cells was transplanted into recipient mice via tail vein injection.

### Flow cytometry

Hematopoietic cells were collected from peripheral blood and bone marrow of the wild type, *Icsbp^−/−^, Lyn^−/−^*, and CML mice, and red blood cells were lysed with NH_4_Cl red blood cell lysis buffer (pH 7.4). The cells were washed with PBS, and stained with B-220 for B cells, Gr-1 for neutrophils, Lin^-^Sca-1^+^c-Kit^+^ for hematopoietic stem cells, Hoechst blue for DNA, and 7AAD/Annexin V for apoptosis analysis. Lineage cocktail includes anti-CD5 (rat IgG2a), anti-CD11b (rat IgG2a), anti-CD45R (B220) (rat IgG2a), anti-7-4 (rat IgG2a), anti-Gr-1 (Ly-6G/C, rat IgG2a), and anti-Ter-119 (rat IgG2b).

### In vitro culture of leukemia stem cells

Bone marrow cells isolated from CML mice were culture in vitro in the presence of stemspan SFEM, SCF, IGF-2, TPO, heparin and α-FGF as described previously [9].

### Drug treatment

Imatinib was dissolved in water directly at a concentration of 10 mg/ml, and administered orally by gavage in a volume less than 0.5 ml (100 mg/kg body weight, twice a day), beginning at day 8 after induction of CML and continuing until the morbidity or death of leukemic mice. Zileuton (Critical Therapeutics) was dissolved in water. The drugs were given orally by gavage (300 mg/kg, twice a day), beginning at 8 days after induction of CML, and continuing until the morbidity or death of the mice with leukemia. IPI-504 was dissolved in a solution containing 50 mM citrate, 50 mM ascorbate, 2.44 mM EDTA, pH 3.3.

### RNA extraction and real-time PCR

Total mRNA was isolated from HSCs or LSCs sorted by FACS, and cDNA was synthesized using the Ovation-Pico cDNA synthesis method. Real-time PCR (RT-PCR) was carried out to detect expression of *β-actin*, *Icsbp* and *Lyn* with the primers for *β-actin* (forward: GGGGTGTTGAAGGTCTCAAA; reverse: ACTGGGACGACATGGAGAAG), *Icsbp* (forward: CAGATCCTCCCTGACTGGTG; reverse: CATCCGGCCCATACA ACTTA), and *Lyn* (forward: ACCCTTATGATGGCATCCAC; reverse: GAAAGCTCCTGCACTGT TGC).

### Data analysis and statistical methods

Statistical analyses were performed by *t* test or GraphPad Prism v5.01 software for Windows (GraphPad Software, San Diego, CA USA).

## Supporting Information

Figure S1 **LSK^−^ cells cannot be produced from Lin^−^cKit^+^Sca1^−^ (LS^−^K) cells.** CD45.1^+^LS^−^K cells were sorted from bone marrow of mice by FACS and transplanted into CD45.2 recipient mice (1×10^6^ per mouse). CD45.1 cells in peripheral blood (A) or bone marrow (B) of recipient mice were monitored with time.
(PDF)

Figure S2 **Induction of CML in secondary recipient mice.** (A) Bone marrow cells were transduced with BCR-ABL-GFP or empty vector (as a control) to induce primary CML. Bone marrow cells from primary CML mice were transplanted into secondary recipient mice (1×10^6^ cells per mouse). The mice developed CML, as shown by FACS analysis of peripheral blood (A) and died (B).
(PDF)

Figure S3 **DNA microarray analysis shows downregulation of** ***Icsbp*** **expression by BCR-ABL in LSCs and partial restoration of** ***Icsbp*** **expression in the absence of** ***Alox5*** **.**
(PDF)

Figure S4 **Real-time PCR analysis shows downregulation of** ***Icsbp*** **expression by BCR-ABL in LSCs and restoration of** ***Icsbp*** **expression in the absence of** ***Alox5*** **.** *: *p*<0.05.
(PDF)

Figure S5 **Loss of** ***Icsbp*** **causes a decrease of LSK^−^ cells in aged mice.** Bone marrow cells from aged WT and *Icsbp−/−* mice (24 weeks) were collected and the percentages and numbers of LSK and LSK^−^ cells in bone marrow were measured by FACS. **: *p*<0.01.
(PDF)
