# Supplementary figures and images for: LSK Derived LSK– Cells Have a High Apoptotic Rate Related to Survival Regulation of Hematopoietic and Leukemic Stem Cells
Source: PLoS One. 2012 Jun 4;7(6):e38614. doi: 10.1371/journal.pone.0038614 (PMC3366951; doi:10.1371/journal.pone.0038614)

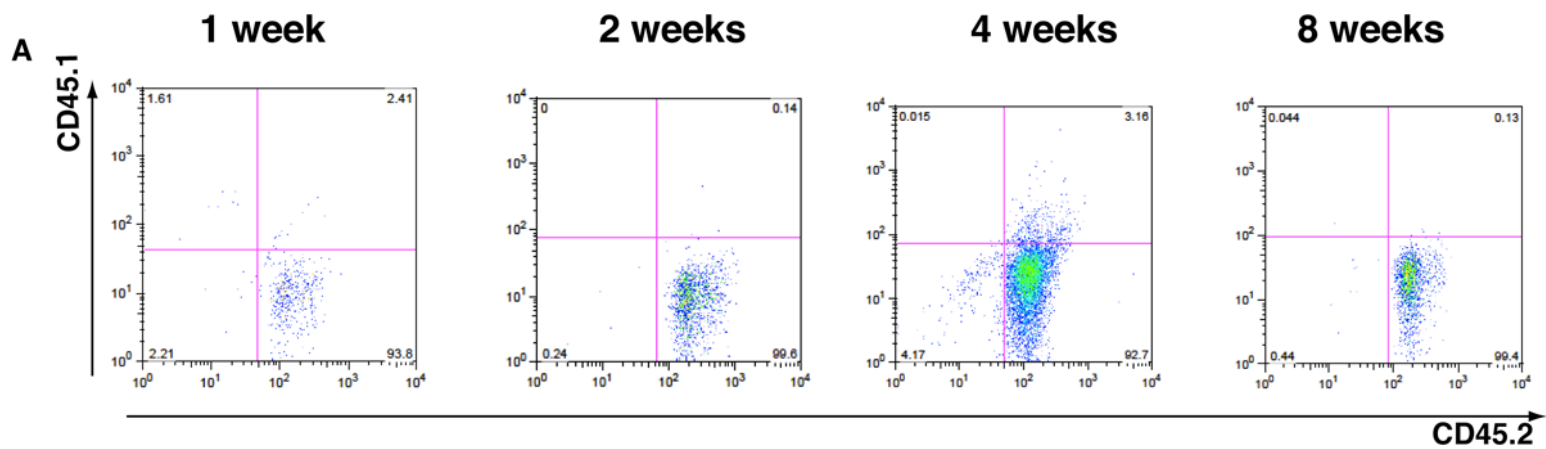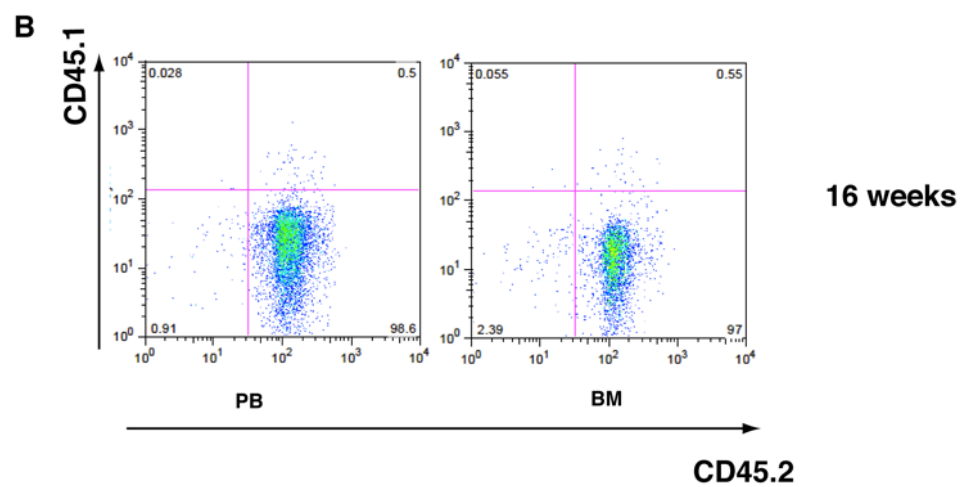

Supplement: Figure S1 — LSK− cells cannot be produced from Lin−cKit+Sca1− (LS−K) cells. CD45.1+LS−K cells were sorted from bone marrow of mice by FACS and transplanted into CD45.2 recipient mice (1×10^6^ per mouse). CD45.1 cells in peripheral blood (A) or bone marrow (B) of recipient mice were monitored with time. (PDF) [file pone.0038614.s001.pdf]

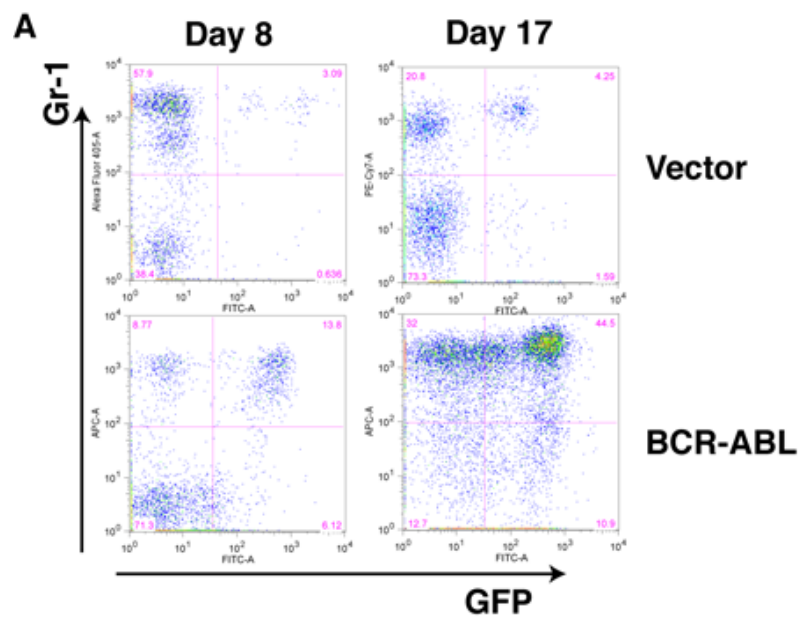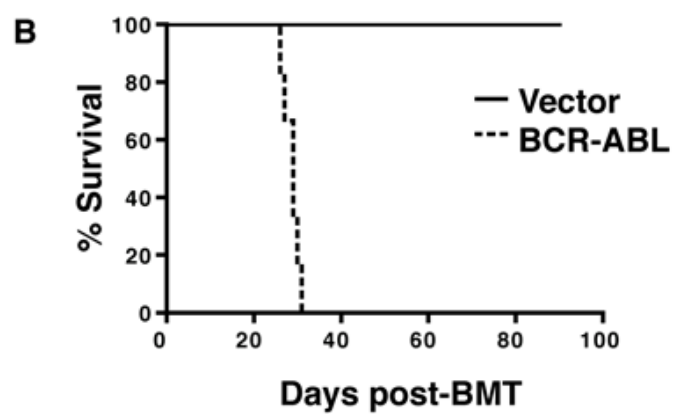

Supplement: Figure S2 — Induction of CML in secondary recipient mice. (A) Bone marrow cells were transduced with BCR-ABL-GFP or empty vector (as a control) to induce primary CML. Bone marrow cells from primary CML mice were transplanted into secondary recipient mice (1×10^6^ cells per mouse). The mice developed CML, as shown by FACS analysis of peripheral blood (A) and died (B). (PDF) [file pone.0038614.s002.pdf]

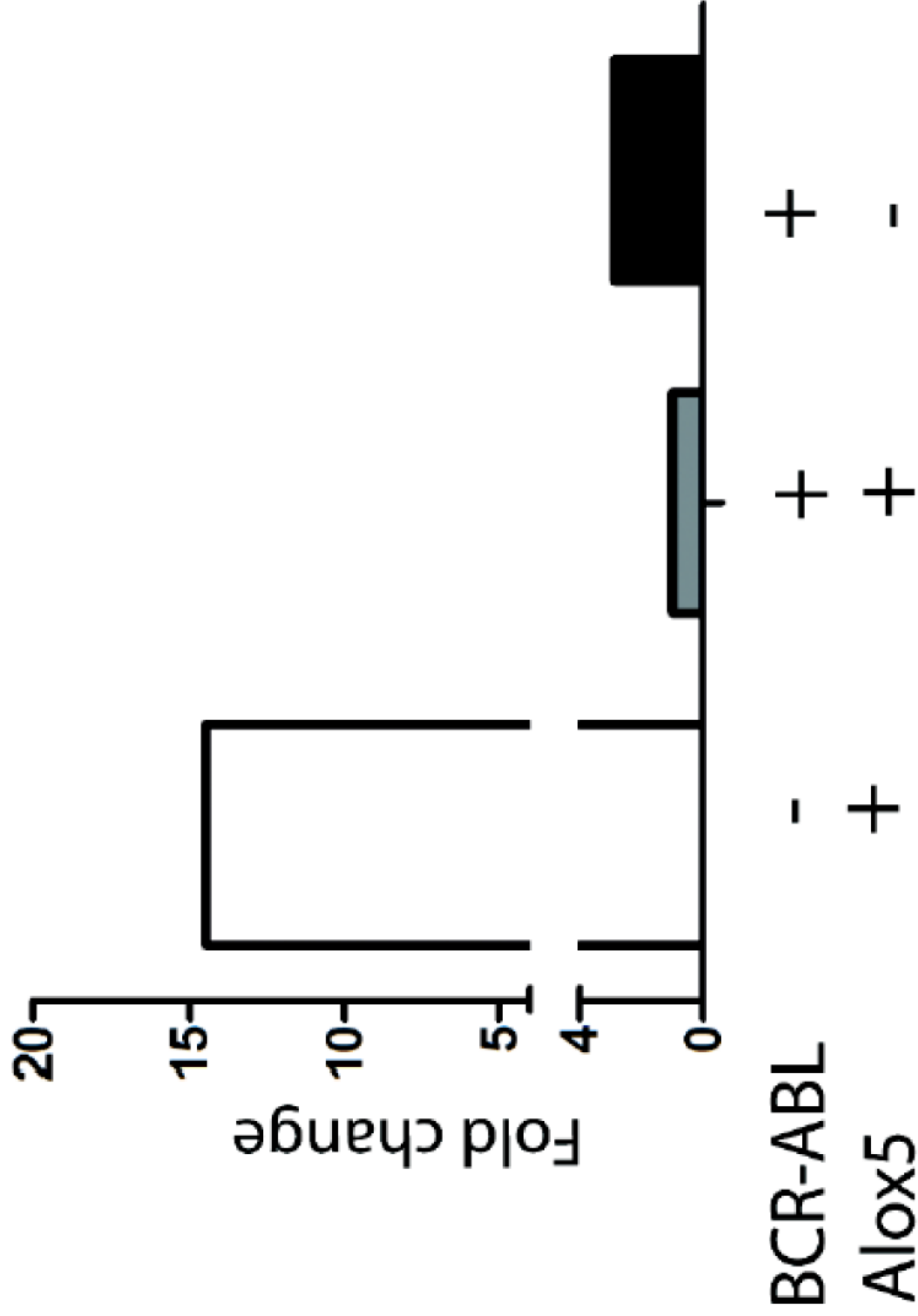

Supplement: Figure S3 — DNA microarray analysis shows downregulation of Icsbp expression by BCR-ABL in LSCs and partial restoration of Icsbp expression in the absence of Alox5 . (PDF) [file pone.0038614.s003.pdf]

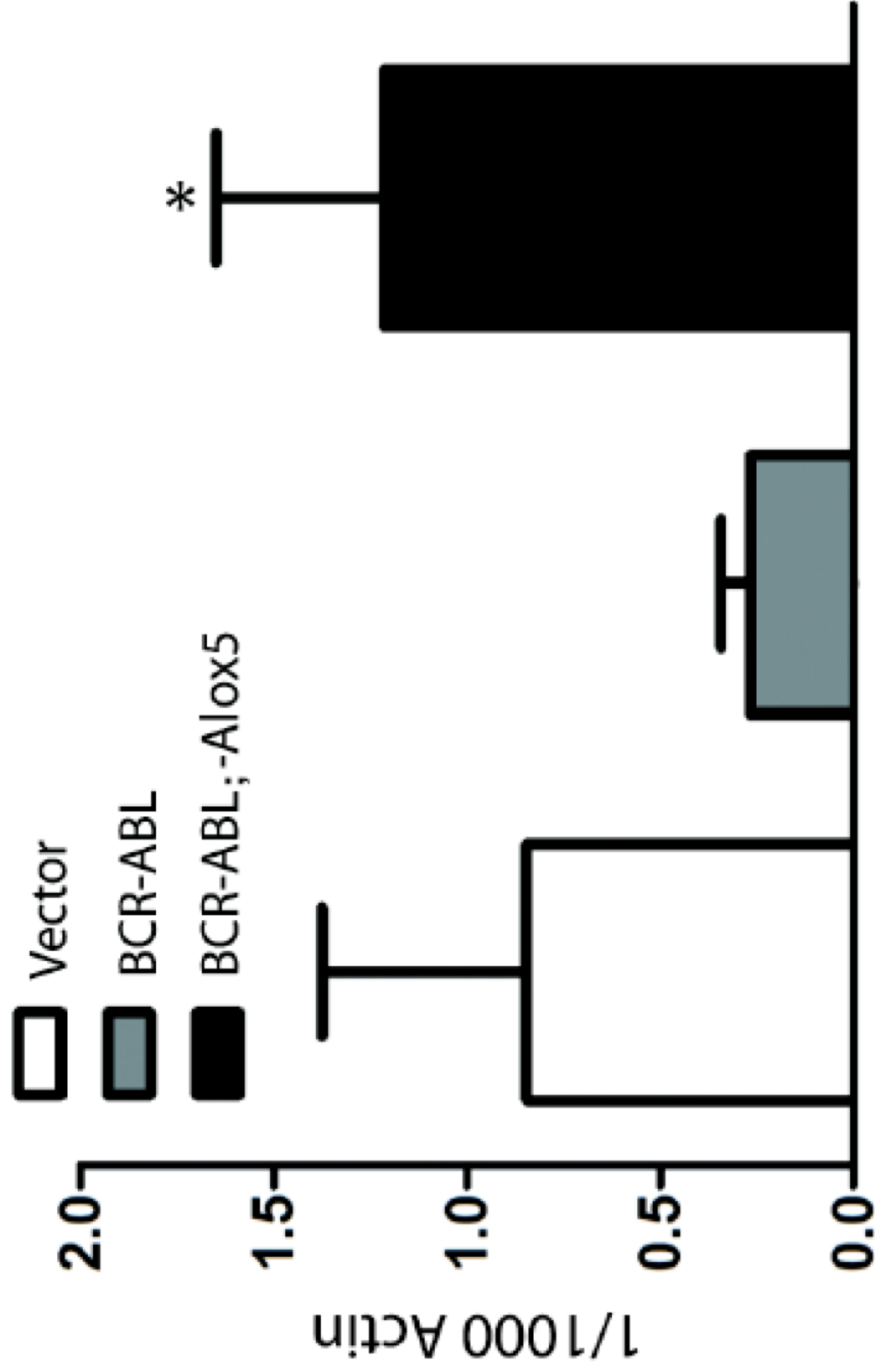

Supplement: Figure S4 — Real-time PCR analysis shows downregulation of Icsbp expression by BCR-ABL in LSCs and restoration of Icsbp expression in the absence of Alox5 . *: p<0.05. (PDF) [file pone.0038614.s004.pdf]

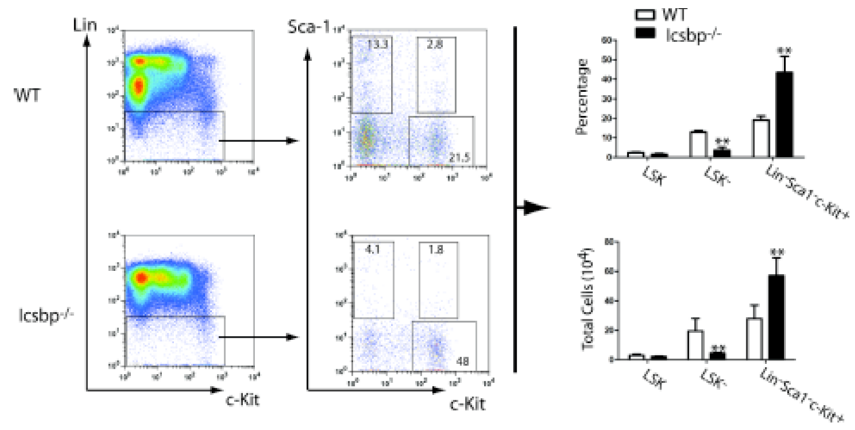

Supplement: Figure S5 — Loss of Icsbp causes a decrease of LSK− cells in aged mice. Bone marrow cells from aged WT and Icsbp−/− mice (24 weeks) were collected and the percentages and numbers of LSK and LSK− cells in bone marrow were measured by FACS. **: p<0.01. (PDF) [file pone.0038614.s005.pdf]
